# Agroinoculation of *Grapevine Pinot Gris Virus* in tobacco and grapevine provides insights on viral pathogenesis

**DOI:** 10.1371/journal.pone.0214010

**Published:** 2019-03-19

**Authors:** Giulia Tarquini, Giusi Zaina, Paolo Ermacora, Francesca De Amicis, Barbara Franco-Orozco, Nazia Loi, Marta Martini, Gian Luca Bianchi, Laura Pagliari, Giuseppe Firrao, Emanuele de Paoli, Rita Musetti

**Affiliations:** 1 Department of Agriculture, Food, Environmental and Animal Sciences, University of Udine, Udine, Italy; 2 ERSA, Plant Protection Service, Pozzuolo del Friuli (UD), Italy; Louisiana State University, UNITED STATES

## Abstract

The Grapevine Pinot Gris disease (GPG-d) is a novel disease characterized by symptoms such as leaf mottling and deformation, which has been recently reported in grapevines, and mostly in *Pinot gris*. Plants show obvious symptoms at the beginning of the growing season, while during summer symptom recovery frequently occurs, manifesting as symptomless leaves. A new *Trichovirus*, named *Grapevine Pinot gris* virus (GPGV), which belongs to the family *Betaflexiviridae* was found in association with infected plants. The detection of the virus in asymptomatic grapevines raised doubts about disease aetiology. Therefore, the primary target of this work was to set up a reliable system for the study of the disease in controlled conditions, avoiding interfering factor(s) that could affect symptom development. To this end, two clones of the virus, pRI::GPGV-vir and pRI::GPGV-lat, were generated from total RNA collected from one symptomatic and one asymptomatic *Pinot gris* grapevine, respectively. The clones, which encompassed the entire genome of the virus, were used in *Agrobacterium-*mediated inoculation of *Vitis vinifera* and *Nicotiana benthamiana* plants. All inoculated plants developed symptoms regardless of their inoculum source, demonstrating a correlation between the presence of GPGV and symptomatic manifestations. Four months *post inoculum*, the grapevines inoculated with the pRI::GPGV-lat clone developed asymptomatic leaves that were still positive to GPGV detection. Three to four weeks later (*i*.*e*. ca. 5 months *post inoculum*), the same phenomenon was observed in the grapevines inoculated with pRI::GPGV-vir. This observation perfectly matches symptom progression in infected field-grown grapevines, suggesting a possible role for plant antiviral mechanisms, such as RNA silencing, in the recovery process.

## Introduction

A grapevine disease consisting of leaf mottling and deformation has been recently reported in northeast Italy and Slovenia[1]. Infected plants show symptoms of stunting, chlorotic mottling, and leaf deformation at the beginning of the growing season, while during summer leaves frequently appear symptomless.

The disease was detected for the first time in *Pinot gris*, so that the disorder is also called “*Grapevine Pinot gris* disease” even though it was later identified in other varieties, such as *Traminer*, *Tocai* (*Friulano*) and *Glera* [2].

The aetiology of the *Grapevine Pinot gris* disease (GPG-d) is still questioned: in 2012 a new virus, named *Grapevine Pinot Gris virus* (GPGV), was identified in diseased grapevines in Trentino-Alto Adige (northeast Italy) [3], but its presence could not be directly correlated to the symptoms because the virus was detected in all symptomatic grapevines but also in plants showing no visible alteration [2–4]. The virus was then detected in grapevines from other Italian regions affected by the disease as well as from other countries, although a number of these wine growing regions have never reported symptoms of the disease [4].

Phylogenetic studies have been conducted with the aim of correlating symptomatic or asymptomatic phenotypes with specific genetic features [1,5,6], however, no univocal correlation has ever been demonstrated. Nevertheless, a relationship between plant symptoms and virus titre was reported, revealing that a higher virus titre occurred in plants showing severe symptoms [2,6].

The occurrence of a multitude of different confounding factors (e.g. adverse environmental conditions and/or abiotic stresses, presence of multiple infections, synergistic effects induced by different pathogens), which affect field-grown grapevines by altering their physiology [7], represents a further complication in deciphering GPG-d-associated symptoms, preventing the establishment of a clear correlation between virus presence and diseased plant phenotype [8]. For this reason, field-grown grapevines are not the most suitable material to study GPG-d aetiology.

Thus, we developed a model system to reproduce GPGV infection under controlled conditions avoiding any external factor(s) that may affect plant response and symptom appearance.

Two GPGV isolates were collected from field-grown plants, one from a symptomatic *Pinot gris* grapevine and the other from an asymptomatic plant. Their full-length cDNAs (7.25 Kb) were reconstructed and cloned into a binary vector. Both viral clones, from symptomatic (pRI::GPGV-vir) and asymptomatic (pRI::GPGV-lat) grapevines, were then used in *Agrobacterium*-mediated inoculation experiments, using *Nicotiana benthamiana* and *Vitis vinifera*. *N*. *benthamiana* was chosen because it is commonly regarded as a more convenient model plant than *V*. *vinifera* to study host-pathogen interactions in viral disease [9].

Nevertheless, grapevine is the natural host of GPGV, thus it was crucial to investigate the specific GPGV/grapevine interaction and to clearly demonstrate the disease aetiology.

The results proved the strong reliability of the experimental setup used in this study and provided insights about GPGV and host relationships, demonstrating under our experimental conditions the correlation between the presence of the virus and symptom occurrence, independent of the viral strain.

Further studies are in progress to demonstrate a possible role of recovery in the onset of asymptomatic leaves on infected grapevines at later stages of infection.

## Results

### Symptom description in agroinoculated plants

Before their use in agrodrench experiments, all *V*. *vinifera* plants were tested for the presence of GPGV and viruses and viroids included in the Italian certification program, namely grapevine viruses A and B (GVA, GVB), grapevine fleck virus (GFkV), grapevine leafroll-associated viruses 1, 2, 3 (GLRaV-1, GLRaV-2, GLRaV-3), grapevine fanleaf virus (GFLV), and arabis mosaic virus (ArMV). RT-qPCR assays excluded the presence of GPGV and the viruses listed above. Nevertheless, evidence was found for the presence in all tested grapevines of grapevine rupestris stem pitting-associated virus (GRSPaV), hop stunt viroid (HSVd), and grapevine yellow speckle viroid 1 (GYSVd-1), which are ubiquitous in grapevines [10–12].

Both *N*. *benthamiana* and grapevine plant groups showed symptoms regardless of whether they were agroinfiltrated with the virulent (pRI::GPGV-vir) or latent (pRI::GPGV-lat) clones.

**Fig 1:**
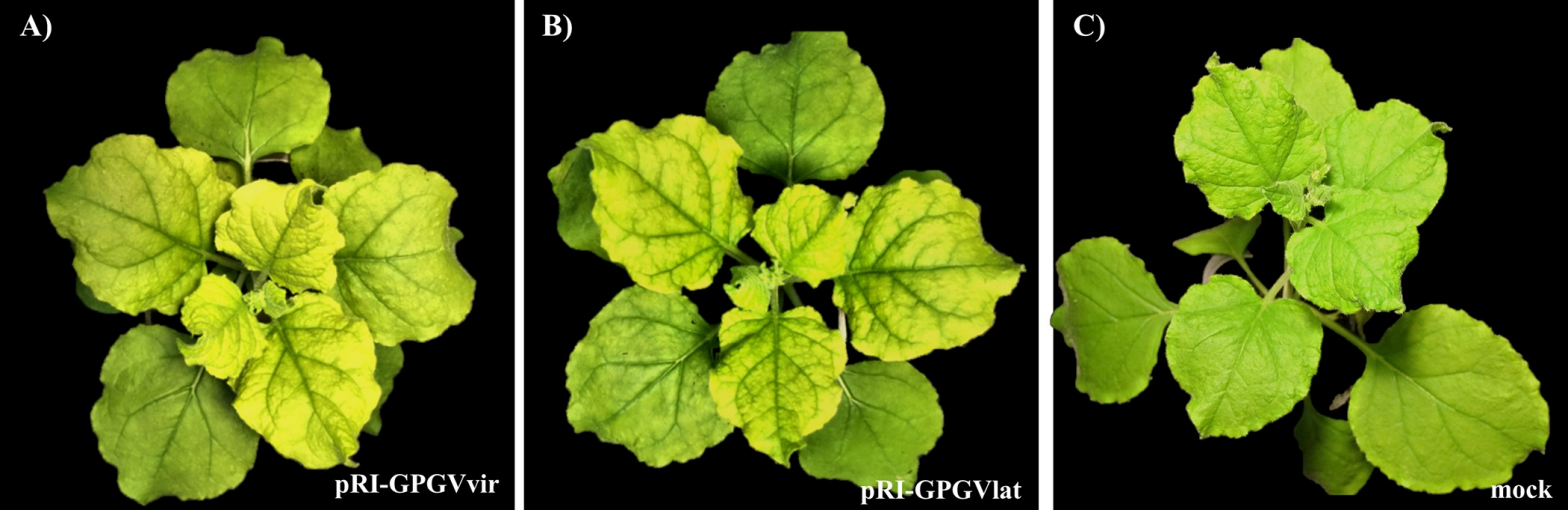
Symptoms observed in *Nicotiana benthamiana* plants agroinfiltrated with clones of *Grapevine Pinot gris* virus. *N*. *benthamiana* plants infected with the virulent (pRI::GPGV-vir, **A**) and latent (pRI::GPGV-lat; **B**) clones of GPGV showing systemic mottling and chlorosis 2 weeks *post inoculum*; **C**) asymptomatic *N*. *benthamiana* inoculated with empty vector (mock).

Two independent agroinfiltration experiments were conducted using *N*. *benthamiana* plants. In both experiments, all plants infiltrated with viral clones exhibited symptoms 2 weeks *post inoculum* (Fig 1A and 1B), such as leaf mottling and widespread chlorosis. No symptoms were observed in mock infiltrated plants (i.e. plants infiltrated with empty vector; Fig 1C). Ten days later, all inoculated plants showed a visible attenuation of symptoms, regardless of the clone used as the inoculum (not shown), and within one month *post inoculum* they became completely asymptomatic.

**Fig 2:**
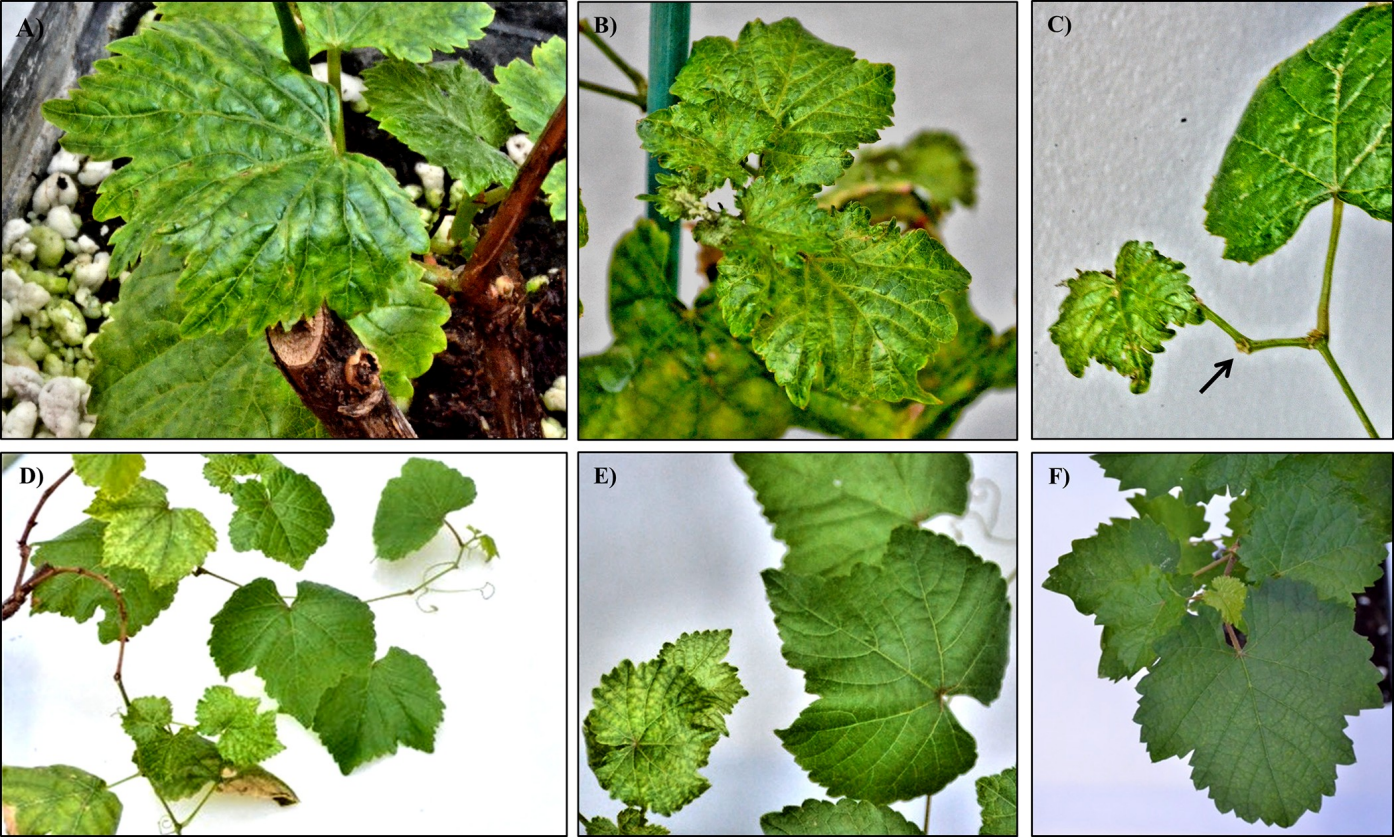
Symptoms observed in *Vitis vinifera* plants agrodrenched with clones of *Grapevine Pinot gris* virus observed 4 months *post inoculum*. **A**) and **B**) chlorotic mottling developed on emerging leaves; **C**) short internode (arrow) displays zigzag growth; **D**) and **E**) *V*. *vinifera* agrodrenched with the latent strain of GPGV showing recovered, lateral branches 5 months *post inoculum*; **F**) asymptomatic *V*. *vinifera*, agrodrenched with empty vector (mock).

Thirteen out of 20 agrodrenched grapevines, (6 inoculated with the virulent and 7 inoculated with the latent clone of the virus) developed severe symptoms 4 months post-inoculation. Two plants (1 inoculated with the virulent and 1 inoculated with the latent clone of the virus) showed visible symptoms 1 week later. Five plants (3 from the virulent and 2 from the latent group) died from drought stress maybe caused by the long submersion period during the root inoculation process. Symptoms were identical to those observed in infected field-grown grapevines [3]: leaf mottling and chlorosis (Fig 2A and 2B), and short internodes with zigzag growth (arrow, Fig 2C). Interestingly, 3 out of 7 plants inoculated with the latent clone recovered from symptoms 4 months *post inoculum*, developing new lateral branches that were completely symptomless (Fig 2D and 2E). Three-four weeks later (*i*.*e*. ca. 5 months after inoculation), recovery from symptoms also occurred in 4 out 8 plants inoculated with the virulent clone. No symptoms were observed in plants infiltrated with the empty vector (mock, Fig 2F).

### Ultrastructural modifications in *Nicotiana benthamiana* and *Vitis vinifera* agroinoculated leaf tissues

TEM observations allowed localization of viral particles and assessment of ultrastructural modifications in leaf tissues from both *V*. *vinifera* and *N*. *benthamiana* agroinoculated plants. For observations, symptomatic leaves located distally from the agroinfiltration point were chosen in *N*. *benthamiana*, whereas leaves showing mild symptoms were chosen in *V*. *vinifera*.

**Fig 3:**
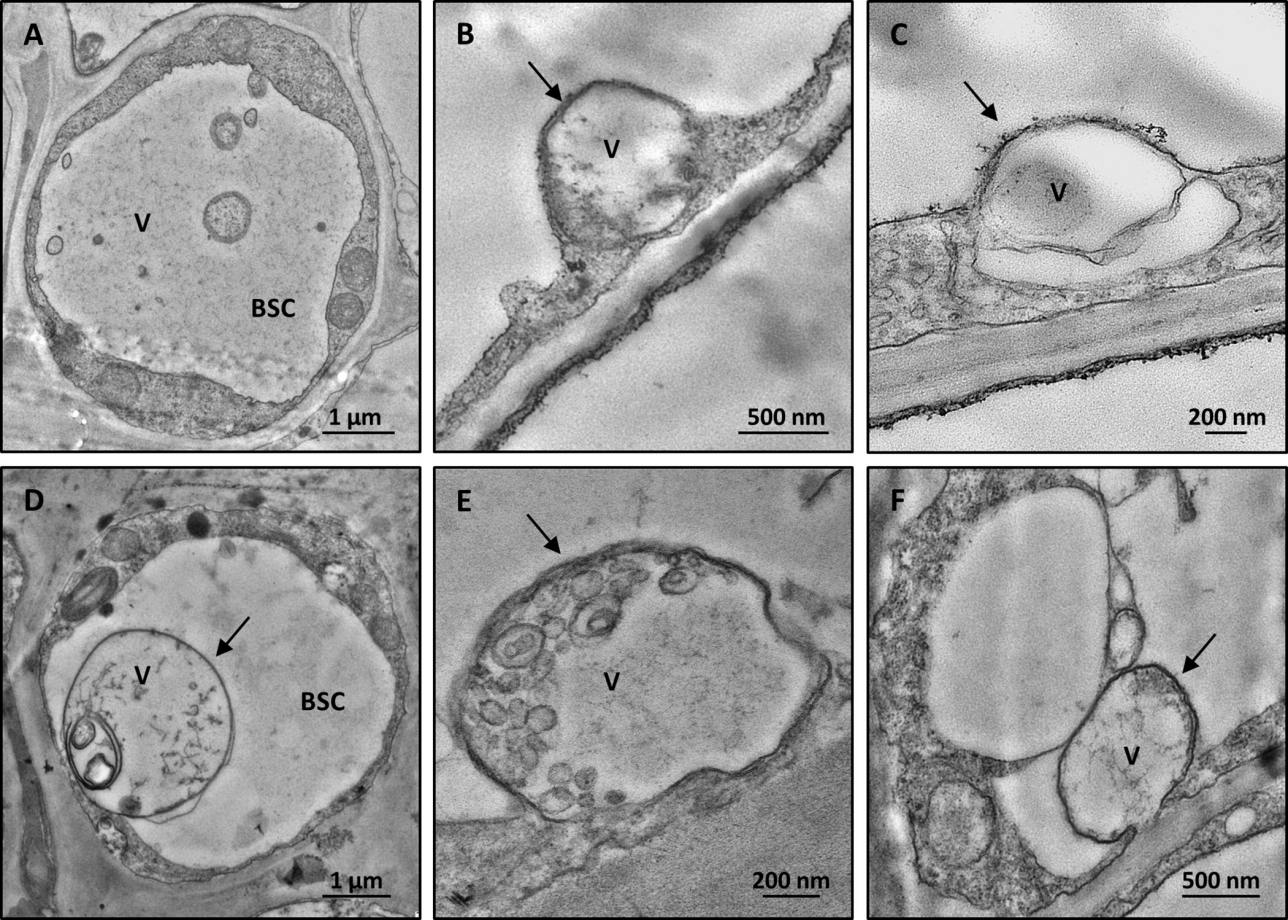
Representative TEM micrographs of leaf tissue from agroinoculated plants. Filamentous flexuous virus-like particles are detected in leaf bundle sheath cells of agroinfiltrated *N*. *benthamiana* (**A, B, C**) and agrodrenched *V*. *vinifera* (**D, E, F**). In the bundle sheath cells, membrane-bound organelles (arrow) are observed in both *N*. *benthamiana* (**B, C**) and *V*. *vinifera* (**D, E, F**) agroinoculated plants. In agrodrenched *V*. *vinifera*, large globular vesicles and filamentous virus-like particles can be clearly detected inside the membrane-bound organelles (D, E, F, arrows). (BSC: bundle sheath cell, V: virus-like particles).

**Fig 4:**
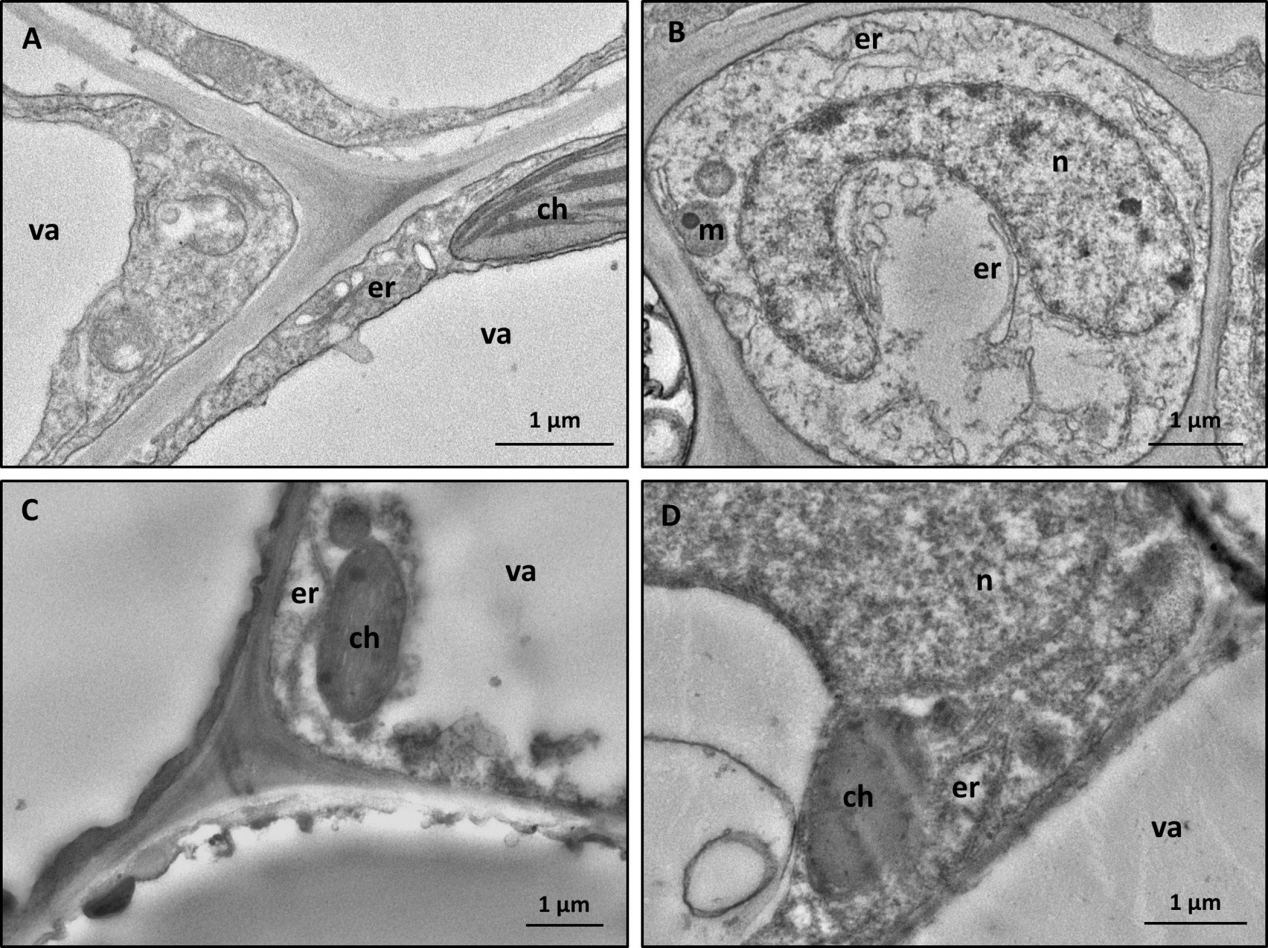
TEM micrographs from leaf tissues of mock-infiltrated plants. Parenchyma cells are well preserved and contain normal-shaped organelles in the leaf tissues of both in *N*. *benthamiana* (**A, B**) and *V*. *vinifera* (**C, D**). (ch: chloroplast, er: endoplasmic reticulum, m: mitochondrion, n: nucleus, va: vacuole).

Leaves of both host species showed the same ultrastructural alterations regardless of the viral clone used for agroinoculation. Filamentous virus-like particles were detected exclusively in the bundle-sheath-cells **(**BSC, *N*. *benthamiana* Fig 3A and *V*. *vinifera* Fig 3D), often inside membrane-bound organelles (*N*. *benthamiana* Fig 3B and 3C and *V*. *vinifera* Fig 3E and 3F). The above-described structures were not observed in control leaf tissues (*N*. *benthamiana*, Fig 4A and 4B, and *V*. *vinifera*, Fig 4C and 4D), which showed cell organelles (endoplasmic reticulum, mitochondria, chloroplasts, nuclei) with normal morphology.

In agroinoculated samples, ultrastructural changes were comparable, for localization and morphology, to those recently reported in field-grown GPGV-infected grapevines [12].

### Detection and quantification of GPGV in *Nicotiana benthamiana* and *Vitis vinifera* agroinoculated plants

The presence of GPGV in *N*. *benthamiana* and *V*. *vinifera* inoculated plants was estimated by RT-qPCR assays using the specific primer GPGV-504 forward and GPGV-588 reverse, as detailed above. *GAPDH* gene was found stably expressed (M-values lower than 0.2) [13] in both grapevine and tobacco systems, so it was used as reference gene for the detection and quantification of GPGV.

**Fig 5:**
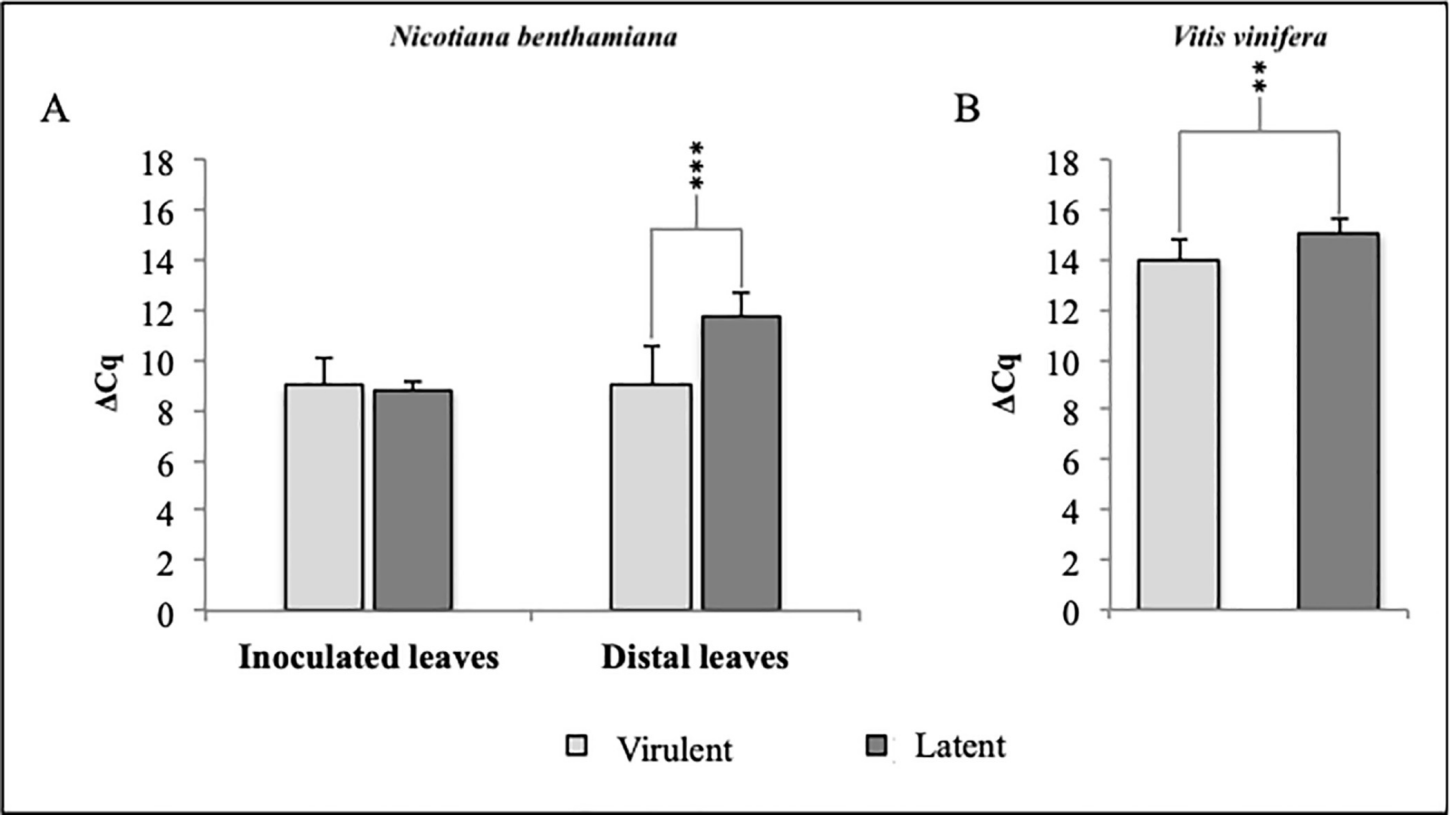
**Viral titre in infected tissues of *N*. *benthamiana* agroinoculated plants (A) and agrodrenched *V*. *vinifera* (B) evaluated 4 months *post inoculum*.** In both species, plants inoculated with the latent GPGV clone show a significantly higher ΔCq value, corresponding to a lower virus titre.

All agroinfiltrated plants (20x2 *N*. *benthamiana* and 20 *V*. *vinifera*) tested positive for GPGV, with Cq values lower than 34. Viral titre was also evaluated, and the mean of ΔCq values, obtained from samples inoculated with the virulent or the latent clone, was compared **(**Fig 5A and 5B**)**.

Lower ΔCq values indicate higher viral concentration in infected tissues [2,6]. Two weeks *post inoculum* the relative viral titre in distal leaves of *N*. *benthamiana* plants inoculated with the virulent or latent clone of GPGV were 9.0 ± 1.4 and 11.7 ± 0.5, respectively (F = 15.86; P = 0.004). On the other hand, the inoculated leaves exhibited the same viral concentration revealing mean values equal to ΔCq of 8.9 ± 1.9 and 8.8 ± 1.6, respectively (F = 0.03; P = 0.87) (Fig 5A). All the data were collected from two independent agroinfiltration experiments.

Four months *post inoculum V*. *vinifera* agrodrenched-plants also showed significant differences in their relative viral titre. Plants inoculated with the virulent clone of GPGV revealed a significantly higher viral concentration (ΔCq 13.9 ± 1.2) than those inoculated with the latent clone (ΔCq 15.1 ± 1.1), (F = 3.8; P = 0.07), (Fig 5B). The two plants (1 inoculated with the virulent and 1 inoculated with the latent clone of the virus) that developed late symptoms showed the highest Cq values (ΔCq 14.88 and 15.50, respectively), suggesting a lower viral concentration in infected tissues [2,6].

Moreover, asymptomatic leaves from the newly developed asymptomatic branches, which tested positive to GPGV, showed Cq values similar to those of symptomatic leaves collected from the same plants.

### Immunocytochemical identification of GPGV in agroinoculated plants

**Fig 6:**
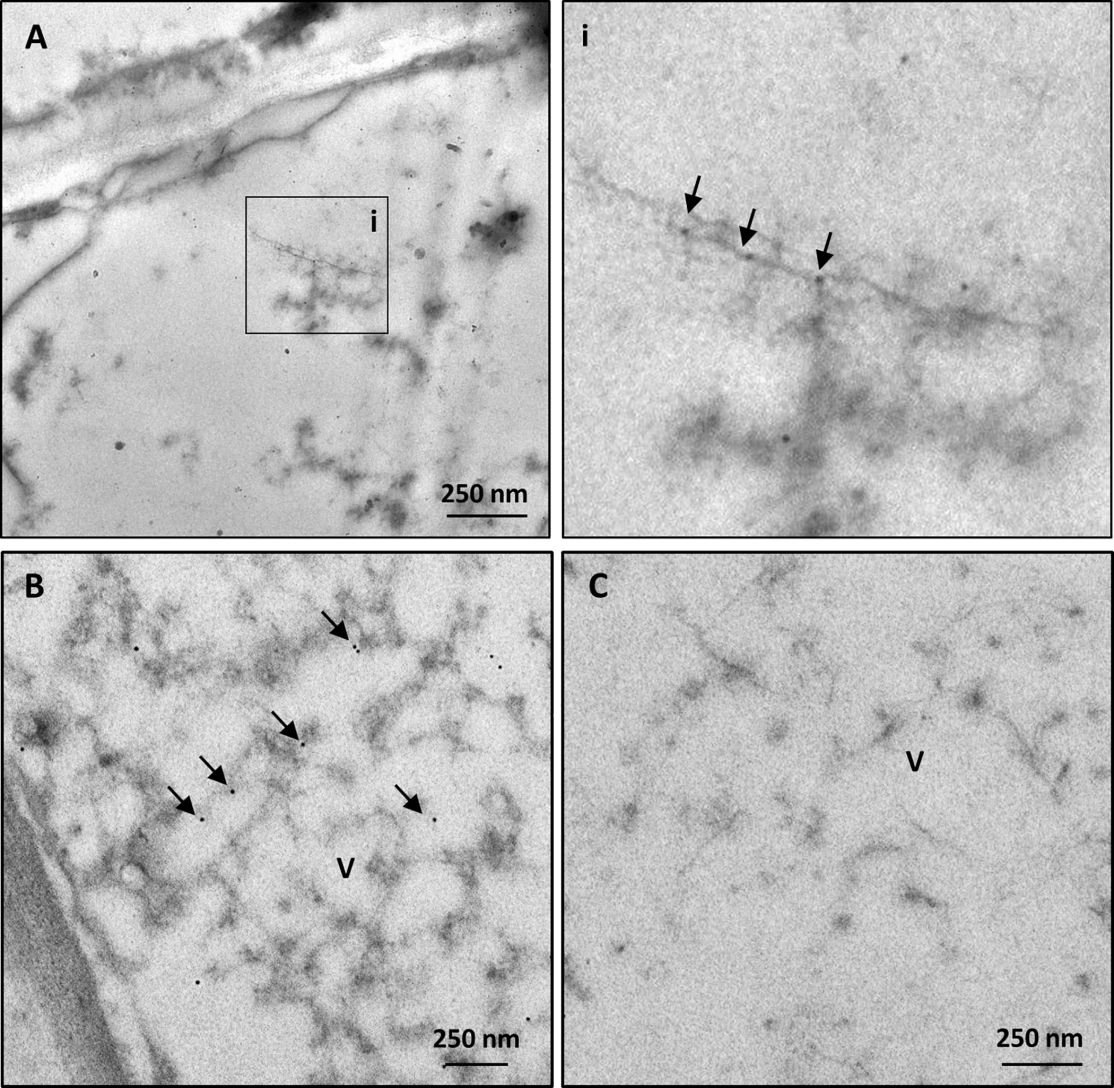
Representative TEM micrographs of immunogold-labelled agroinoculated plants. In samples incubated with a 1:10 dilution of primary rabbit polyclonal antibody (Pab) against GPGV-coat protein and a 1:50 dilution of secondary gold-conjugated antibody, gold (arrows) is visible in the bundle sheath cells of agroinoculated plants in association with the filamentous particles and in their proximity (**A, B**). In inset (i), area of interest of A is magnified. Label does not occur in infected samples incubated with buffer alone (**C**). (V; virus).

Immunocytochemical analyses revealed positive reaction of anti-GPGV-CP Pab with the virus-like filamentous structures observed in BSCs of agroinoculated plants. Using 1:10 dilutions of Pab and 1:50 of GAR, the gold label signal was detected exclusively in proximity to the filamentous particles (Fig 6A and 6B). No label was observed in agroinoculated plants incubated with normal goat serum alone (NGS, Fig 6C).

## Discussion

The lack of correlation between virus presence and symptom occurrence has always been a crucial issue in the study of GPG-d. In vineyards, asymptomatic plants that do not exhibit any visible alteration were frequently found beside symptomatic grapevines [2]. For this reason, the disease aetiology is currently a subject of debate.

Field-grown grapevines are difficult to investigate, being subjected to biotic and abiotic stresses [7,8] and being potentially affected by multiple infections (i.e. by various viruses and viroids) that can interact with each other, exhibiting synergistic [14] or antagonistic effects [15].

To evaluate plant responses to GPGV infection in terms of symptom development, ultrastructural modification, virus titre and systemic spread, and limiting the influence of external factors as much as possible, we attempted to reproduce GPG-d in controlled conditions. For this purpose, the entire genome of two GPGV isolates was cloned.

The construction of full-length cDNA clones represents an essential and powerful technique to study the pathogenesis of RNA viruses, revealing the intriguing cross-talk that mediates viral infection [16–18]. In fact, despite the difficulties involved in the cloning of full-length viral cDNA, this approach has greatly improved the study of virus/host interactions through the analysis of phenotypic effects in infected plants [19–21], also providing an excellent tool for reverse-genetic studies on plant viruses [16,22]. However, before this work, no full-length cDNA clone of GPGV had been made available.

The full-length cDNA clones were agroinoculated into *N*. *benthamiana* and *V*. *vinifera* plants, allowing us to investigate virus/plant interactions in both the model (*N*. *benthamiana*) and the natural (*V*. *vinifera*) hosts. In such experimental systems both GPGV clones induced visible symptoms in the plant hosts as well as ultrastructural modifications that were identical to those observed in infected field-grown grapevines [12].

The results presented here demonstrated the ability of both cDNA clones to produce infectious, replicating virus units, which were detected as filamentous and flexuous particles within infected tissues. These particles were very similar in shape, size and location to those found in GPGV-infected field-grown grapevines [12]. The difference in virus titre found in agroinoculated leaves of *N*. *benthamiana* compared to the distal leaves revealed a possible difference in the spreading ability of the two GPGV clones used in this study. A lower viral titre in the distal leaves has been associated with a non-efficient systemic spread of virus within infected tissues [23]. Thus, it can be speculated that virulent and latent clones of GPGV may have a different ability to move systemically, which could be associated with specific polymorphisms detected in the movement protein sequence ([1]; Tarquini et al., manuscript under review).

A major finding of this work was that 50% *V*. *vinifera* plants agrodrenched with latent and 50% of those agrodrenched with virulent showed symptom recovery in adult leaves despite still being positive for GPGV. Because the tested plants were grown in a greenhouse, with a maximum temperature of 28° C, the disappearance of symptoms cannot be explained by hot summer temperatures, as reported to occur in other viral diseases in field-grown grapevines [24]. As an attempt to explain this result, we hypothesize that the activation of a plant-mediated RNA silencing mechanism occurred in grapevine [25]. This mechanism relies on biogenesis of viral-derived small-RNAs (vsRNAs), which are able to promote degradation of the complementary viral genome [25]. Activation of the RNA silencing machinery may lead to symptom recovery, i.e. the establishment of a virus-tolerant state within infected tissues, in which plants develop asymptomatic leaves that still contain infectious, replicating viral particles [26–29].

Recovery in GPGV-infected plants could be an explanation for the frequent absence of recordable symptoms in GPGV infected grapevines in the field. Further studies are in progress to demonstrate the induction of recovery in GPGV-infected grapevines via a plant RNA-silencing mechanism [26,30].

## Materials and methods

### Plant material

In this study, thirty self-rooted *Vitis vinifera* cv. *Pinot gris* plantlets and a total of sixty *Nicotiana benthamiana* seedlings (the latter obtained from two independent experiments), were grown as GPGV natural and model hosts, respectively.

Before their use in agrodrench experiments (see paragraph below), all *V*. *vinifera* plantlets were tested with real-time RT-PCR to exclude the presence of GPGV and all the viruses included in the Italian certification program [2,31].

Grapevines that tested negative for the presence of the above-cited viruses were chosen for agrodrench experiments and grown in a hydroponic system as follows: roots were thoroughly washed, surface-sterilized using 1% hydrogen peroxide solution for 30 min and then placed in Hoagland medium [32]. Before being inoculated, plants were maintained for 3 weeks in a greenhouse with temperature and photoperiod replicating typical spring to early summer field conditions (24–25° C max, 15–16° C min, and 13h light/11h dark photoperiod).

*N*. *benthamiana* seedlings were grown in a growth chamber at 21°C and 60% relative humidity (RH) under a 16h light/8h dark photoperiod for about 3 weeks before agro-infiltration. After inoculation, both *N*. *benthamiana* and *V*. *vinifera* plants were kept in the same conditions.

### Construction of full-length cDNA clones of *Grapevine Pinot gris virus* (GPGV)

The GPGV fvg-Is12 (accession MH087443) and fvg-Is15 (accession MH087446) isolates (Tarquini et al., manuscript under review) were chosen for this study to represent an isolate from a symptomatic grapevine, hereafter named “virulent” (fvg-Is12) and an isolate from a symptomless grapevine named “latent” (fvg-Is15). Their partial cDNA (about 7.1 Kb) was obtained by 5’- RACE (Tarquini et al., manuscript under review).

The cDNA was then amplified by long-distance PCR with specific primers (pRI101_BamHI_5’cDNA forward and Internal-3’cDNA reverse as in Table 1). To complete the missing 3’-end of the viral genome, the amplified products were purified and assembled with two synthetic fragments **(**Table 1**)** designed from the KR528581.1 viral reference sequence [33], using a one-step Gibson assembly procedure according to the manufacturer’s protocol (New England BioLabs, UK). The full-length cDNA was inserted into the BamHI/SacI-digested pRI101-AN DNA binary vector (Clontech Laboratories–Takara BIO, USA, Inc.), following the protocol provided with the In-Fusion HD cloning kit (Clontech Laboratories, USA).

**Table 1:** List of DNA primers used in this study.

Name	Primer sequences		Primer application
pRI101_BamHI_5’cDNA	For.	ACCCCGGGGGTACCGGATCCTAAAACACGTAAGGTTGAATCTAGC	LD-PCR
Internal-3’cDNA reverse	Rev.	GCATTAGTCTTTTGCTTCTCACTTTCGACATGAAAAAGC	
Internal_3cDNA	-	GCAAAAGACTAATGCTATCACGGCTTCGGGGGAGAGTGCATTTAGTAT GTAGTTATATGTTTTATATAATAATAAAGTCT	3’-end synthetic fragments
3cDNA_EcoRI_pRI101	-	TATATAATAATAAAGTCTCATAGGAGCACGTAACTTCTTAATGTCTAC GTAAGTTTGTTTTAATTAATTTTCTTCT GAATCAACAACTCT	
GPGV-504	For	GAATCGCTTGCTTTTTCATG	RT-qPCR
GPGV-588	Rev	CTACATACTAAATGCACTCTCC	
VvGAPDH	For	GCTGCTGCCCATTTGAAG	
VvGAPDH	Rev	CCAACAACGAACATAGGAGCA	
NbGAPDH	For	AGCTCAAGGGAATTCTCGATG	
NbGAPDH	Rev	AACCTTAACCATGTCATCTCCC	

pRI101_BamHI_5’cDNA and Internal-3’cDNA reverse primers were used to amplify the partial cDNA of GPGV for the Gibson assembly experiments. Synthetic fragment were constructed on reference sequence KR528581.1 [33]to complete the missing 3’-end of the viral genome. RT-qPCR primers were employed to detect and quantify GPGV in the infected tissues of inoculated plants.

The recombinant plasmids, pRI::GPGV-vir and pRI::GPGV-lat, were transformed into NEB Stable Competent *Escherichia coli* cells, following the manufacturer's protocol (New England BioLabs, UK) and selected on LB agar plates containing 50 μg/ml kanamycin (Sigma Aldrich, USA, Inc). Plasmids with the expected molecular size (17.7 Kb) were selected and purified with a PureYield Plasmid Miniprep System (Promega, USA) according to the manufacturer’s protocol and sequenced with Illumina MiSeq technology (IGA Technology Services, Italy). The sequence-validated plasmids were introduced into *Agrobacterium tumefaciens* strain LBA4404 by electroporation (Takara Bio, USA, Inc).

### Agrobacterium-mediated inoculation of GPGV clones in *Nicotiana benthamiana* and *Vitis vinifera* plants

Agroinoculation was performed both in *N*. *benthamiana* and *V*. *vinifera* to compare symptom expression, ultrastructural alterations and virus titre in plants inoculated with *Agrobacterium* harbouring either the pRI::GPGV-vir or the pRI::GPGV-lat clones. A single colony of *A*. *tumefaciens* strain LBA4404 carrying the appropriate viral clone was inoculated into 5 ml of LB medium supplemented with 50 μg/ml kanamycin, and grown overnight at 30°C with constant shaking. Cells were harvested by centrifugation at 3000 x g for 10 min at 4°C, and resuspended in infiltration medium (10 mM MES pH 5.8 and 200 μM acetosyringone) with an OD_600_ adjusted to 0.5 and 1.0 for *N*. *benthamiana* and *V*. *vinifera*, respectively. The undersides of fully expanded leaves of 3-week old *N*. *benthamiana* plants were infiltrated using a needleless 2 ml syringe, while *V*. *vinifera* virus-free plants were inoculated through the roots, using a modified version of the agrodrench technology described by Muruganantham and co-authors [34]. Briefly, a single plant was transferred into a sterile pot containing 1:10 Agrobacterium inoculum re-suspended in Hoagland’s nutrient solution. The plantlets were kept in the Agrobacterium suspension for 10 days and then transferred into a hydroponic system supplied with Hoagland medium. A total of 30 plants of both species were used: 10 individual plants were tested for the presence of each construct (pRI::GPGV-vir and pRI::GPGV-lat), whereas pRI101-empty vector was inoculated in 10 plants used as negative controls.

Two independent agroinfiltration experiments were carried using *N*. *benthamiana*, *i*.*e*. a total of 60 plants was definitively prepared and evaluated.

All *N*. *benthamiana* and *V*. *vinifera* plants were monitored for symptom expression.

### Conventional transmission electron microscopy

Symptomatic leaves of agroinoculated *N*. *benthamiana* and *V*. *vinifera* plants were collected for ultrastructural analysis 2 weeks and 4 months *post inoculum*, respectively. Distal leaves of *N*. *benthamiana* were collected to observe subcellular modifications, whereas leaves showing typical GPG-d symptoms were chosen for the analyses in *V*. *vinifera*. Segments (3–4 mm in length) of leaf tissues including both vein tissue and surrounding parenchyma cells were fixed in 3% glutaraldehyde, rinsed in 0.15 M phosphate buffer (PB), postfixed in 1% osmium tetroxide in 0.15 M PB for 2 h at 4°C, dehydrated in ethanol and embedded in Epon-Araldite epoxy resin (Electron Microscopy Sciences, Fort Washington, PA, USA) according to the method described by [35]. Ultrathin sections (60–70 nm) of about 20 resin-embedded samples from each transformed or control plants were cut using an ultramicrotome (Reichert Leica Ultracut E ultramicrotome, Leica Microsystems, Wetzlar, Germany) and collected on 200 mesh uncoated copper grids. Sections were then stained with UAR-EMS (uranyl acetate replacement stain) (Electron Microscopy Sciences, Fort Washington, PA, USA) and observed under a PHILIPS CM 10 (FEI, Eindhoven, The Netherlands) transmission electron microscope (TEM), operated at 80 kV, and equipped with a Megaview G3 CCD camera (EMSIS GmbH, Münster, Germany). Five non-serial cross-sections from each sample were analysed.

### RT-qPCR analyses and identification of reference gene

RT-qPCR assays were performed to detect and quantify GPGV in leaf tissues of *N*. *benthamiana* and *V*. *vinifera* plants, both agroinoculated with the infectious clones of the virus and mock infiltrated. To achieve this, both the agroinoculated and the distal leaves from each *N*. *benthamiana* and the distal leaves from each *V*. *vinifera* were collected 2 weeks and 4 months *post inoculum*, respectively, when symptoms, resembling those associated with GPG-d, were clearly evident. From the 20 *N*. *benthamiana* (10 for each independent experiment) and the 10 *V*. *vinifera* plants that had been mock-infiltrated (i.e. infiltrated with empty vector) a single leaf was sampled from each plant and taken as negative control.

In grapevine, leaf material from the newly developed asymptomatic branches was also sampled for virus detection.

Total RNA was extracted from frozen and ground leaf tissues, using Spectrum Plant Total RNA (Sigma Aldrich, USA, Inc) in accordance with the procedure provided in the kit. RNA concentration was determined using a NanoDropND-100 spectrophotometer (NanoDrop Technologies) and its integrity was evaluated by electrophoresis on a 1.2% agarose gel in TBE 0.5X buffer. cDNA was synthesized from 500 ng of total RNA using the recombinant *Moloney Murine Leukemia Virus* reverse transcriptase (MMLV-RT; Promega, USA) according to manufacturer's protocol. Five ng of the resulting cDNA was subjected to qPCR using the specific primers, GPgV504-F and GPgV588-R **(****Table** 1**)**, according to the protocol described by Bianchi and co-authors [2].

The reference gene was individuated comparing, in plants agroinoculated either with virulent or latent GPGV clones, the expression of *GAPDH* (glyceraldehyde-3-phosphate dehydrogenase), *ACT* (actin), *α-EF* (Elongation factor) and *UBIQ10* (polyubiquitin 10) for *V*. *vinifera* and *GAPDH* (glyceraldehyde-3-phosphate dehydrogenase), *PP2A* (Protein phosphatase 2A) and *F-box* (F-box protein) for *N*. *benthamiana* **(**S1 Table**).** The expression stability of the different candidate reference genes was evaluated, using the software program *geNorm* NormFinder [36,37], which indicated *GAPDH* as the most suitable reference gene for both *N*. *benthamiana* and *V*. *vinifera* (S1 Table). Moreover, *GAPDH* as reference gene was previously proposed by other authors for GPGV quantification in infected plants collected in field [2,6].

All reactions were performed at least in duplicate using a CFX96 real-time system (Bio-Rad, Hercules, CA, USA) and amplification data were analysed with CFX Manager Software 2.0 (Bio-Rad). To allow comparability between assays, the baseline threshold was always set to 300 RFU (relative fluorescence units) and samples were scored positive for GPGV when threshold cycle (Cq) values were < 34 [2]. Relative quantification of the virus in inoculated plants was calculated with the comparative Cq (2^-ΔΔCq^) method, using the sample with the smallest amount of the virus as a control [6]. Statistical analyses were performed with the *AnalystSoft (StatPlus v*.*6)* software using one-way ANOVA and *Tukey-Kramer* multiple comparisons test as the post hoc test. A P value < 0.05 was considered statistically significant.

### Immunocytochemical detection of GPGV in leaf tissues of agroinoculated plants

An immunogold labelling experiment was carried out to provide further evidence about the presence of GPGV in agroinoculated plants. One distal leaf was collected from each of five *N*. *benthamiana* and *V*. *vinifera* agroinoculated plants (total of 5 leaves per species), ensuring that they were coeval, had similar shape and showed the typical symptoms of GPG-d [12]. Similarly, single distal leaves from five mock plants (plants inoculated with empty vector) were also collected and tested as negative controls.

The experiment was performed according to the protocol reported by Tarquini and co-authors [12]. Samples were cut into small portions (6–7 mm in length), fixed 1 h in 0.2% glutaraldehyde, rinsed in 0.05 M PB pH 7.4, and dehydrated in graded ethanol series (25, 50, 75%, 30 min for each step) at 4°C. After 1 h of the final 100% ethanol step, the samples were infiltrated in a hard-grade London Resin White (LRW, Electron Microscopy Sciences, Fort Washington, PA, USA) / ethanol 100% mixture in the proportion 1:2 for 30 min, followed by LRW/ethanol 2:1 for 30 min, and 100% LRW overnight at room temperature (with a change 1 h after the start of the infiltration). The samples were embedded in beem capsules (Electron Microscopy Sciences, Fort Washington, PA, USA) using fresh LRW containing benzoyl peroxide 2% (w/w) according to manufacturer’s protocol, and polymerized for 24 h at 50°C.

Several ultrathin sections (60–70 nm) from a total 40 LRW embedded samples from *N*. *benthamiana* and *V*. *vinifera* were cut using an ultramicrotome (Reichert Leica Ultracut E ultramicrotome, Leica Microsystems, Wetzlar, Germany) and collected on carbon/formvar-coated 400 mesh nickel grids (Electron Microscopy Sciences, Fort Washington, PA, USA). Non-specific binding sites were blocked by placing grids carrying the sections on droplets of blocking solution, containing 0.05 M Tris-buffered saline (TBS), pH 7.6, and 1:30 normal goat serum (NGS) for 1 hour. Grids were then incubated overnight with primary rabbit polyclonal antibody (Pab) against GPGV coat protein (Bioreba AG, Reinach, Switzerland). The Pab was diluted 1:10 in 0.05 M TBS, pH 7.6 containing 1:30 NGS. Control grids were incubated only in TBS/NGS solution without primary antibody. All grids were washed five times in 0.05 M TBS (for 3 min each one), treated for 1 h with secondary goat anti-rabbit antibody conjugated with colloidal 10 nm gold particles (GAR 10; EM GAR G10 BBI solutions, Cardiff, UK) diluted 1:50 in TBS, and then washed again as described above.

Sections were fixed in 2% glutaraldehyde for 5 min, then in 1% OsO_4_ for 15 min. After staining with Uranyl Acetate Replacement Stain (UAR-EMS, Electron Microscopy Sciences, Hatfield, PA), samples were observed under TEM, as reported above. Five non-serial cross-sections from each sample were analysed.

## Supporting information

S1 Table S1 Table shows the list of primers used for reference gene identification. (DOCX)
