# Supplementary material for: Agroinoculation of Grapevine Pinot Gris Virus in tobacco and grapevine provides insights on viral pathogenesis
Source: PLoS One. 2019 Mar 19;14(3):e0214010. doi: 10.1371/journal.pone.0214010 (PMC6424481; doi:10.1371/journal.pone.0214010)
Supplement: S1 Table — (DOCX) [file pone.0214010.s001.docx]

**S1 Table. List of primers used for reference gene identification.**

|  | **Sequence** | **Reference** | **M-value** |
| --- | --- | --- | --- |
| *Vitis vinifera* | | | |
| GAPDH | For: GCTGCTGCCCATTTGAAG  Rev: CCAACAACGAACATAGGAGCA | Bianchi *et al.,* 2015 | 0.1234 |
| ACT | For: TTTTGTTCTGCTCACGCATC  Rev: GTAGCCCTCTTCGGACGTAA | unpublished | 0.1811 |
| EF | For: TTTGCTGTTCGTGACATCCCG  Rev: GCTTCCTCTGTTGAGCTCC | unpublished | 0.1301 |
| UBIQ10 | For: CCAAGATCCAGGACAAGGAA  Rev: GAAGCCTCAGAACCAGATGC | Santi *et al.,* 2013 | 0.1234 |
| *Nicotiana benthamiana* | | | |
| GAPDH | For: AGCTCAAGGGAATTCTCGATG  Rev: AACCTTAACCATGTCATCTCCC | Liu *et al.,* 2012 | 0.1954 |
| PP2A | For: GACCCTGATGTTGATGTTCGCT  Rev: GAGGGATTTGAAGAGAGATTTC | Liu *et al.,* 2012 | 0.1364 |
| F-box | For: GGCACTCACAAACGTCTATTTC  Rev: ACCTGGGAGGCATCCTGCTTAT | Liu *et al.,* 2012 | 0.2137 |

GAPDH (glyceraldehyde-3-phosphate dehydrogenase); ACT (actin); α-EF (Elongation factor); UBIQ10 (polyubiquitin 10); PP2A (Protein phosphatase 2A); F-box (F-box protein).
